# AI-Driven Digital Pathology: Deep Learning and Multimodal Integration for Precision Oncology

**DOI:** 10.3390/ijms27010379

**Published:** 2025-12-29

**Authors:** Hyun-Jong Jang, Sung Hak Lee

**Affiliations:** 1 Department of Physiology, CMC Institute for Basic Medical Science, College of Medicine, The Catholic University of Korea, Seoul 06591, Republic of Korea; hjjang@catholic.ac.kr; 2 Department of Hospital Pathology, Seoul St. Mary’s Hospital, College of Medicine, The Catholic University of Korea, Seoul 06591, Republic of Korea

**Keywords:** artificial intelligence, deep learning, digital pathology, foundation model, precision oncology, radiology

## Abstract

Pathology is fundamental to precision oncology, offering molecular and morphologic insights that enable personalized diagnosis and treatment. Recently, deep learning has demonstrated substantial potential in digital pathology, effectively addressing a wide range of diagnostic, prognostic, and biomarker-prediction tasks. Although early approaches based on convolutional neural networks had limited capacity to generalize across tasks and datasets, transformer-based foundation models have substantially advanced the field by enabling scalable representation learning, enhancing cross-cohort robustness, and supporting few- and even zero-shot inference across a wide range of pathology applications. Furthermore, the ability of foundation models to integrate heterogeneous data within a unified processing framework broadens the possibility of developing more generalizable models for medicine. These multimodal foundation models can accelerate the advancement of pathology-based precision oncology by enabling coherent interpretation of histopathology together with radiology, clinical text, and molecular data, thereby supporting more accurate diagnosis, prognostication, and therapeutic decision-making. In this review, we provide a concise overview of these advances and examine how foundation models are driving the ongoing evolution of pathology-based precision oncology.

## 1. Introduction

Recent advances in deep learning (DL) have enabled computational models to extract clinically meaningful patterns from histopathology whole-slide images (WSIs). A growing body of work has demonstrated that morphological features embedded in routine hematoxylin and eosin (H&E) tissue slides contain latent signals associated with molecular phenotypes, tumor microenvironment interactions, and patient prognosis [1]. For example, DL models have been shown to predict key molecular alterations such as microsatellite instability (MSI), Epstein–Barr virus (EBV) status, and specific driver mutations directly from WSIs [2,3]. Such findings suggest that histopathology may serve not only as a diagnostic endpoint, but also as a rich source of surrogate biomarkers for precision oncology, particularly in settings where molecular testing is costly, time-consuming, or unavailable.

Early approaches to computational pathology predominantly relied on convolutional neural networks (CNNs) and multiple-instance learning (MIL) frameworks to handle the gigapixel scale and heterogeneous morphology of WSIs [4]. While these methods achieved promising performance, their generalizability across institutions and cancer subtypes remained limited, partly due to domain shift in staining and slide preparation. Recently, the emergence of vision transformers (ViTs) and self-supervised pretraining has reshaped the field, enabling models to learn more scalable and transferable representations from large WSI datasets. Methods such as large scale ViT-based pathology encoders and hierarchical WSI representation learning have substantially improved robustness and cross-cohort performance [5,6].

Based on this emerging trend, a new generation of pathology foundation models has demonstrated strong zero-shot and few-shot learning capability across diverse tasks [7,8,9]. These models are trained on tens of millions of slide patches or slide-text pairs, allowing them to capture contextual and morphological signals that were previously inaccessible to smaller, task-specific models. As a result, pathology is transitioning from series of task-specialized classifiers toward unified representation models that can serve as general feature extractors for diagnosis, prognosis, and biomarker prediction.

Meanwhile, radiology has also undergone a related transformation with the development of vision–language models that leverage paired datasets of radiology images and clinical reports. Many models align image features with related medical reporting to support visual question answering (VQA), report generation, and structured reasoning [10,11,12]. The parallel progress in radiology and pathology foundation models has laid the groundwork for multimodal foundation models that link morphological, imaging, and clinical text information, enabling more holistic characterization of disease and more informed therapeutic decision-making.

In this review, we first summarize how foundation models have been applied to medical image analysis, with a particular focus on pathology and radiology. We then discuss how multimodal foundation models can expand the role of digital pathology in advancing precision medicine.

## 2. Key Advantage of Foundation Model

A foundation model is a large-scale, pre-trained model that learns generalizable representations from broad and diverse data, enabling adaptation to a wide range of downstream tasks with minimal task-specific training [13]. To appreciate the advantages of foundation models, it is informative to compare them with conventional DL approaches.

**Figure 1:**
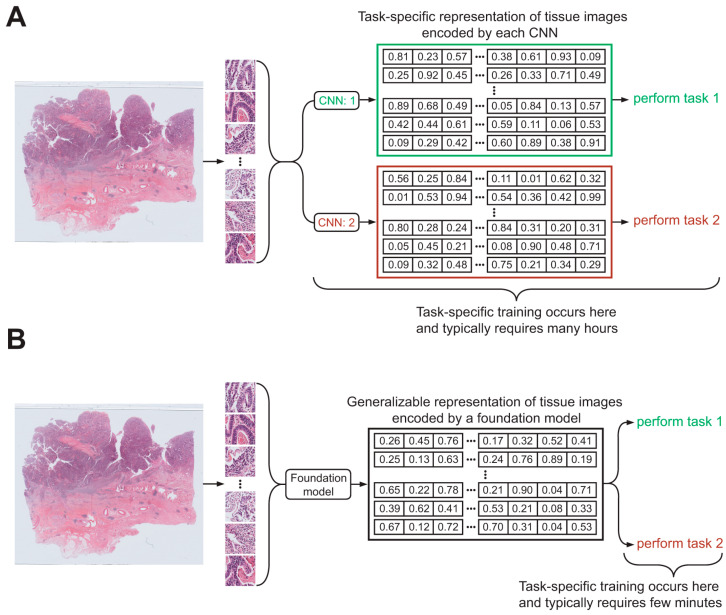
Schematic illustration highlighting the differences between CNN-based and foundation model-based approaches for WSI classification. (**A**) CNN models; (**B**) Foundation model.

**Figure 2:**
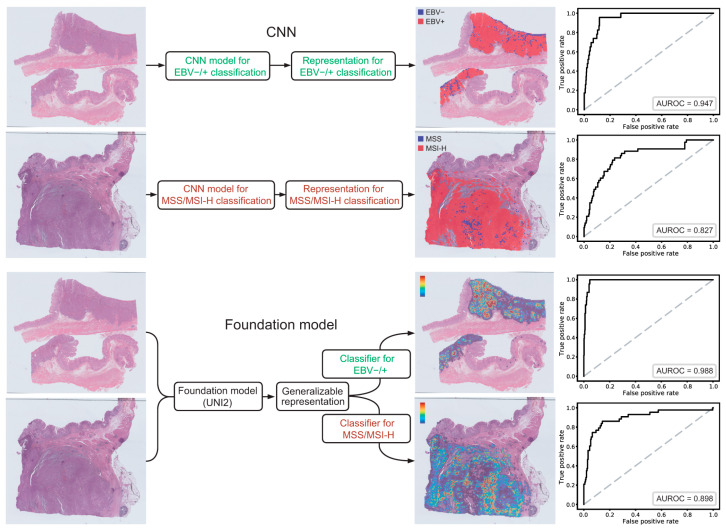
Comparison of CNN-based and foundation model-based classifiers trained to predict EBV status and MSI status in gastric cancer. In both tasks, classifiers built upon foundation model achieved higher performance. The data presented are unpublished results from our laboratory. For CNN-based models, Inception v3 was trained on 360 × 360-pixel tissue image patches extracted at 20× magnification. For the pathology foundation model, UNI2 [5] was employed to extract patch-level feature embeddings, which were subsequently aggregated and classified using a clustering-constrained-attention multiple-instance learning approach [4]. AUROC: area under the receiver operating characteristic curves.

In the early stage of computational pathology, CNN-based models were primarily developed to address specific diagnostic or predictive tasks from WSIs. For instance, CNNs can learn to discriminate MSI status or various genetic mutations from WSIs of gastric cancer [14,15]. However, because the learned representations in CNNs are task-specific, the resulting feature space is not interoperable or reusable across different downstream problems. As a result, each new task typically necessitates developing and training an entirely separate model, which increases computational burden, reduces data efficiency, and limits scalability across diverse clinical applications (Figure 1A). By contrast, foundation models are trained on large-scale, heterogeneous WSI datasets to capture broad, context-rich, and biologically meaningful representations of tissue morphology. These representations serve as shared, task-agnostic feature backbones, enabling diverse downstream tasks—such as classification, biomarker prediction, prognosis estimation, and slide-level retrieval—to be learned with minimal training cost (Figure 1B). This paradigm improves both data efficiency and generalizability by allowing a common representation to be reused across multiple tasks. Figure 2 demonstrates that foundation model-based approaches outperform CNN-based models across multiple tasks when trained on the same WSI datasets.

## 3. Conventional Deep Learning Models for Pathology

Early works in pathology demonstrated that DL can predict genetic mutations, molecular subtypes, gene and protein expression, treatment response, and prognosis directly from WSIs, primarily using CNN-based models. Before the advent of foundation models, the primary paradigm in computational pathology involved training supervised CNN models, such as Inception or ResNet architectures, on patch-level data to identify specific phenotypes. These studies were pivotal in establishing that routine H&E slides contain latent visual signals reflecting the underlying molecular biology of tumors, effectively turning histopathology into a surrogate for more expensive and time-consuming molecular assays. In this section, key findings from representative studies are briefly summarized, with detailed study characteristics provided in Table 1.

A significant area of exploration has been the prediction of driver mutations. Coudray et al. published a seminal study demonstrating that a DL model could classify non-small cell lung cancer subtypes and predict mutations in key driver genes [2]. This work provided early definitive evidence that specific mutations induce subtle morphological changes recognizable by computer vision algorithms. Extending this concept, Schaumberg et al. successfully trained a model to predict *SPOP* mutations in prostate cancer and validated it on an external cohort, proving that these morphological signatures could generalize across datasets [16].

Researchers subsequently tested applicability across diverse cancer types. Fu et al. developed a pan-cancer pipeline to distinguish tissue types and predict mutations, though performance varied significantly by gene and cancer type [17]. Similarly, Kather et al. highlighted that while some mutations like *TP53* were consistently predictable, others were context dependent [18]. Addressing data characteristics, Jang et al. demonstrated that tissue preparation methods and dataset size significantly influence performance in colorectal cancer mutation prediction [19]. Furthermore, Loeffler et al. showed that DL could outperform human experts in detecting *FGFR3* mutations from bladder cancer histology [20].

DL has also been applied to complex biomarkers such as tumor mutational burden (TMB). Xu et al. demonstrated the feasibility of distinguishing TMB status in bladder cancer [21]. Jain and Massoud refined this in lung adenocarcinoma using a multi-scale approach, suggesting relevant information is distributed across cellular and architectural levels [22]. Similarly, Shimada et al. showed that DL could accurately predict TMB status in colorectal cancer, offering a cost-effective alternative to manual counting or sequencing [23].

The classification of intrinsic molecular subtypes has been another major focus. Couture et al. trained a model to discriminate between basal-like and non-basal-like subtypes in breast cancer [24]. This capability is particularly valuable as it enables molecular subtyping from standard histology without additional testing. Addressing domain shifts, Sirinukunwattana et al. employed domain adversarial training to classify four consensus molecular subtypes (CMS1 to 4) in colorectal cancer, disentangling robust signals from cohort-specific noise [25]. Hong et al. extended this to endometrial cancer, accurately predicting molecular subtypes including copy-number high tumors [26].

DL models have also shown the ability to quantify gene and protein expression levels, effectively performing “virtual staining.” Sha et al. predicted PD-L1 expression from H&E images in non-small cell lung cancer [27]. This application highlights the potential of AI to standardize subjective clinical assessments, such as visual evaluation of immunostained tissue slides. Moving beyond single proteins, Schmauch et al. predicted RNA-seq profiles for thousands of genes across various cancers, finding that spatial attention maps correlated with actual expression patterns [28]. He et al. advanced this using spatial transcriptomics as ground truth, demonstrating links between local morphological patterns and transcriptional programs [29].

Finally, DL models have demonstrated utility in predicting patient prognosis and treatment response. Bychkov et al. pioneered outcome prediction in colorectal cancer using tissue microarrays, outperforming visual risk assessment by pathologists [30]. In glioma, Mobadersany et al. integrated histology with genomic data to improve survival prediction compared to single modalities [31]. Large-scale validation by Skrede et al. solidified clinical relevance, using an ensemble model to stratify colorectal cancer patients into prognostic groups [32]. Saillard et al. reported similar success in hepatocellular carcinoma, predicting survival independently of standard clinical variables [33]. Regarding therapy response, Johannet et al. demonstrated that DL could predict immunotherapy responses in melanoma, particularly when analyzing lymph node metastases [34].

Beyond the studies discussed in this section, numerous additional works have focused on addressing digital pathology tasks using conventional DL approaches. Several review articles have provided comprehensive overviews of these early efforts [35,36,37,38].

In summary, these conventional DL approaches established the fundamental evidence that histopathology images are dense data carriers of various clinical information. By leveraging CNNs to associate morphology with genomic, transcriptomic, and clinical labels, these studies paved the way for the current era of foundation models, which aim to overcome the task-specific limitations and lack of generalizability that characterized these earlier, yet groundbreaking, efforts.

## 4. Representative Foundation Models for Pathology

Recent progress in computational pathology has been driven by the development of foundation models trained on large-scale WSI datasets. These models aim to learn task-agnostic, transferable representations of tissue morphology that can be efficiently adapted to diverse downstream applications, including cancer subtype classification, biomarker prediction, grading, and prognosis prediction. Unlike traditional CNN-based models, which are trained separately for each task, foundation models provide a shared morphological embedding space that supports zero-shot, few-shot, and lightweight fine-tuning paradigms.

In Section 4 and Section 5, we review selected foundation models that represent pivotal milestones in the field. The models were chosen based on their architectural innovation, the scale of pretraining data, and their demonstrated ability to set new state-of-the-art performance across downstream tasks.

Among pathology foundation models, several representative approaches illustrate distinct strategies in representation scale, learning methodology, and multi-resolution integration (Table 2). Here, we briefly review seven representative pathology foundation models—CTransPath, Pathology Language-Image Pretraining (PLIP), Hierarchical Image Pyramid Transformer (HIPT), Virchow, UNI, Clinical Histopathology Imaging Evaluation Foundation (CHIEF), and GigaPath—and summarize their key characteristics. In contrast to conventional CNN models, which are typically designed for specific tasks, these foundation models are capable of supporting a wide range of downstream tasks (Table 3).

CTransPath is a large-scale hybrid model of CNN and transformer specifically designed for computational pathology, leveraging self-supervised hierarchical contextual learning that captures both local cellular morphology and broader tissue organization [39]. The model is pretrained on more than 15 million pathology image patches from diverse cancer types, using a semantically relevant contrastive learning objective. It can be applied to a wide range of downstream tasks, including patch retrieval, patch classification, weakly supervised WSI classification, mitosis detection, and gland segmentation. CTransPath has also served as an important benchmark for many subsequent pathology foundation model architectures.

To incorporate linguistic supervision, PLIP employs a Contrastive Language-Image Pretraining (CLIP)-style image–text contrastive learning framework trained on 208,414 paired histology image tiles and natural-language descriptions collected from medical Twitter [40]. CLIP is a contrastive learning framework that aligns representations obtained from image and text encoders by bringing matching image–text pairs closer together while pushing non-matching pairs apart. By aligning visual representations with linguistic diagnostic descriptors, PLIP captures clinically meaningful semantic structures and demonstrates strong zero-shot performance in differentiating tissue subtypes.

To address the limitation of local context in patch-based models, HIPT introduces a multi-level transformer architecture that progressively aggregates cell-level, patch-level, and region-level representations to form slide representations [6]. This hierarchical design enables the model to retain both local cellular detail and global tissue context, improving performance for cancer subtyping and survival prediction.

More recently, the scale of pretraining dataset has expanded substantially. For example, Virchow leverages large-scale self-supervised learning based on DINOv2 with a ViT-H architecture, trained on 1,488,550 WSIs derived from 119,629 patients [9]. This model learns high-capacity morphological embeddings with strong performance across diverse tumor types and demonstrates robust transferability in various molecular biomarker prediction tasks.

UNI has been introduced as a universal histopathology representation model trained on large-scale multi-institution tiles, emphasizing cross-cohort robustness and serving as a broadly transferable feature backbone for downstream computational pathology tasks [5]. It showed strong few-shot learning capability for multiple tissue classification tasks and molecular biomarker prediction tasks.

CHIEF, a large-scale weakly-supervised foundation framework pretrained on 60,530 WSIs across 19 anatomical sites and utilizing 44 TB of data, achieved substantial gains (up to ~36.1%) over task-specific DL methods in cancer cell detection, tumour origin classification, molecular profiling and prognostic prediction across 32 independent slide sets from 24 hospitals, demonstrating strong robustness to domain shift [7]. CHIEF integrates patch-level features to generate global whole-slide level representations by combining unsupervised pretraining on tile images with weakly supervised pretraining at the whole-slide level.

Finally, GigaPath also extends the representation scope to the whole-slide level, constructing slide embeddings that preserve spatial and architectural relationships across tissue compartments [8]. By learning slide-level context end-to-end, GigaPath supports downstream prognostic modeling and clinical decision-making tasks that require global tissue interpretation. It demonstrated state-of-the-art performance across nine cancer subtyping tasks and seventeen pathomics tasks.

These foundation models rely on several established pretraining paradigms, including self-supervised contrastive learning, DINOv2-style distillation, weakly supervised WSI-level learning, and CLIP-based vision–language alignment. Distinct pretraining paradigms entail critical trade-offs in efficiency, cost, and interpretability. Self-supervised contrastive learning, exemplified by CTransPath, excels at learning discriminative patch-level features but often lacks global context and requires large batch sizes for stability. In contrast, knowledge distillation frameworks like DINOv2 (e.g., Virchow, UNI) demonstrate superior cross-cohort generalizability and representation quality through student–teacher interactions, yet they demand immense computational resources and hundreds of millions of patches. Weakly supervised WSI-level learning, as utilized in CHIEF and GigaPath, offers higher data efficiency for slide-level tasks by aggregating context directly from diagnostic labels, though this increases architectural complexity. Meanwhile, CLIP-based vision–language alignment (e.g., PLIP) uniquely addresses the ‘black-box’ problem by providing superior interpretability and zero-shot capabilities through text alignment. Because no single paradigm is universally superior, future developments must balance these performance gains against the practical constraints of computational infrastructure and data availability in clinical settings.

## 5. Radiology, Multimodal, and Generalist Foundation Models

Radiology has been a particularly fertile domain for the development of foundation models because medical images in this field are routinely accompanied by structured reports. This natural pairing of image and text provides a rich supervisory signal for learning clinically meaningful joint representations. Early radiology models focused on CNN-based classifiers trained on labeled image datasets, such as CheXpert [41] and MIMIC-CXR [42], to detect abnormalities or predict disease severity. However, these models could not leverage the semantic richness of radiology reports.

The introduction of vision–language models addressed this limitation by aligning image features with textual descriptions. Methods such as BioViL [10], MedCLIP [43] and PMC-CLIP [44] demonstrated that contrastive learning between images and text reports can yield generalizable image–text representations suitable for downstream tasks such as image retrieval, classification, and VQA.

More recently, large-scale image–text foundation models built upon large language model (LLM)-vision encoder integration have emerged. To generate reports from chest X-ray images, Large Language and Vision Assistant for Radiology (LLaVA-Rad) introduced a domain-specific, instruction-tuned model optimized for radiology workflows, demonstrating superior performance in radiology VQA, report interpretation, and structured report generation tasks [12].

Beyond chest X-rays, researchers have begun developing general-purpose radiology foundation models capable of handling diverse imaging modalities and tasks. A notable example is RadFM, a recently introduced radiology foundation model trained on web-scale multimodal data [45]. RadFM adopts a unified ViT-based architecture for both 2D and 3D inputs and is designed to process text inputs interleaved with 2D or 3D medical scans, enabling a wide range of radiology tasks—including diagnosis, report generation, and VQA.

Other models are pretrained on diverse medical image–text pairs collected from a wide range of biomedical literature. As a result, they can analyze various image modalities commonly found in medical publications, including radiology and pathology images. Models such as LLaVA-Med [11] and Med-Flamingo [46] extend vision–language models with instruction tuning, enabling interactive question-answering, structured reasoning, and report summarization across multiple medical image modalities.

Integrating LLMs with vision encoders has also enabled pathology foundation models to incorporate linguistic descriptions, allowing them to generate interpretable explanations of WSIs. By combining a pathology-specific vision encoder with a LLM, PathChat can perform a variety of language-based pathology tasks, including answering diagnostic questions [47]. PRISM2 [48] and Transformer-based pathology Image and Text Alignment Network (TITAN) [49] can not only perform classification and biomarker-prediction tasks but also generate slide-level diagnostic dialogues, as they are trained on WSIs paired with clinical diagnostic reports. Collectively, these vision–language pathology foundation models enable interactive, dialogue-based interpretation of WSIs, bringing conversational pathology analysis closer to practical use.

Recent efforts have advanced toward generalist medical foundation models capable of handling diverse medical data types simultaneously [50,51]. In BiomedGPT, heterogeneous multimodal data encompassing EKG, endoscopy, pathology, radiology, ultrasound, and accompanying textual information are integrated to construct a generalist model capable of interpreting and responding to diverse medical conditions [52]. Table 4 and Table 5 provide a comparative summary of the representative foundation models discussed above.

Multimodal DL approaches are not limited to foundation model-based methods. A wide range of other approaches have also been actively developed to integrate multimodal data within unified frameworks. Comprehensive overviews of these important contributions can be found in several existing review articles on multimodal DL [53,54].

## 6. Challenges and Limitations

While recent foundation models have begun to incorporate a broader spectrum of data modalities, the development of truly multimodal generalist foundation models that integrate heterogeneous biomedical data—including WSIs, radiology images, clinical text, genomics, and laboratory data—into a unified representation space remains a distant goal. Training such a comprehensive model remains infeasible at present, as no curated dataset integrating all these modalities is currently available.

Although data scarcity remains a critical bottleneck, a more fundamental difficulty lies in achieving meaningful semantic alignment across heterogeneous modalities, such as histomorphology, genomics, radiology, and clinical text, which differ substantially in scale, structure, and noise characteristics. Naïve fusion strategies risk diluting modality-specific signals or introducing spurious correlations that undermine clinical reliability. Emerging approaches, including weakly paired multimodal learning, intermediate representation alignment, and task-driven partial fusion, offer promising alternatives to fully shared embedding spaces. However, these strategies remain at an early stage of development and have yet to demonstrate robust generalization across institutions and disease contexts.

Beyond these challenges, several additional limitations are inherent to the nature of foundation models themselves. First, the issue of ‘hallucination’ in multimodal vision–language foundation models remains a significant safety concern. Generative models, such as PathChat or LLaVA-Med, may produce plausible but factually incorrect diagnostic reports. In the high-stakes domain of oncology, such errors are unacceptable. Rigorous validation frameworks and human-in-the-loop systems are therefore indispensable. Second, the ‘black-box’ nature of DL is exacerbated in foundation models. While attention maps in CNNs provided some level of interpretability, the complex, multi-layered attention mechanisms in billion-parameter transformers are far more difficult to interpret. Lastly, since the performance of foundation models is heavily dependent on the quality and diversity of pretraining data, addressing dataset bias to prevent disparities in diverse patient populations remains a critical priority for the field.

In addition to algorithmic hurdles, translating pathology foundation models into real-world clinical deployment poses substantial practical challenges. Inter-institutional variability in tissue processing, staining protocols, and scanner hardware remains a major source of performance degradation when models are applied across hospitals. In addition, the computational and storage demands associated with large-scale WSI processing present nontrivial barriers for routine clinical adoption. Inference latency and system integration further complicate deployment in time-sensitive diagnostic workflows. Regulatory considerations, including model validation, auditability, and compliance with evolving medical device regulations, remain insufficiently addressed in most research studies. Finally, the lifecycle management of foundation models—including monitoring for data drift, periodic revalidation, and updating pretrained representations—introduces operational complexities that extend beyond conventional research settings. Together, these factors contribute to a gap between research-level validation and clinical-grade implementation, underscoring the need for deployment-oriented evaluation frameworks in future studies.

## 7. Conclusions

Although early CNN-based approaches were limited to a relatively narrow range of tasks, transformer-based foundation models have substantially expanded the applicability of DL in pathology, enabling a variety of downstream tasks through few-shot and even zero-shot learning. More recently, foundation models have begun to integrate multiple modalities of medical data—including pathology, radiology, and textual descriptions—into a shared representation space, supporting intelligent tasks such as report generation and VQA.

These advances are expected to substantially enhance the capabilities of digital pathology to support holistic patient characterization. Because WSIs encode rich molecular, microenvironmental, and architectural information, pathology-integrated foundation models have the potential to serve as a central hub for precision oncology by linking histomorphology with clinical data, genomic profiles, therapeutic biomarkers, and patient outcomes.

However, to the best of our knowledge, truly generalist medical foundation models—capable of integrating WSIs, radiology images, clinical text, genomics, and laboratory data for comprehensive clinical decision-making—have yet to emerge. Although efforts to incorporate genomic information into imaging data, including histopathology images, are underway, this line of research remains at an early stage [55]. Nevertheless, recent advances in multimodal foundation models are laying the groundwork for such holistic systems, which have the potential to fundamentally transform future medical practice. Continued progress in this direction will require not only larger and more diverse multimodal datasets, but also rigorous evaluation frameworks that ensure safety, fairness, and clinical reliability. With sustained methodological and infrastructural advances, truly generalist medical foundation models may ultimately become central components of future precision diagnostics and clinical decision-support systems.

## Figures and Tables

**Table 1:** Characteristics of conventional deep learning model for pathology.

Reference	Cancer Type	Target	Training Cohort	Base Model	Performance Measure
Coudray et al. [2]	Lung cancer	Various genes	TCGA	Inception v3	AUROC: 0.674~0.845
Schaumberg et al. [16]	Prostate cancer	*SPOP*	TCGA	ResNet-50	AUROC: 0.74
Fu et al. [17]	Various cancers	151 gene cancer pairs	TCGA	Inception v4	AUROC: 0.098~0.972
Kather et al. [18]	Various cancers	Mutations with a prevalence above 2%	TCGA	ShuffleNet	AUROC: 0.55~0.8
Jang et al. [19]	Colorectal cancer	Various genes	TCGA	Inception v3	AUROC: 0.645~0.809
Loeffler et al. [20]	Bladder cancer	*FGFR3*	TCGA	ShuffleNet	AUROC: 0.701
Xu et al. [21]	Bladder cancer	TMB	TCGA	Xception	AUROC: 0.75
Jain and Massoud [22]	Lung cancer	TMB	TCGA	Inception v3	AUROC: 0.92
Shimada et al. [23]	Colorectal cancer	TMB	TCGA+Japan CRC cohort	Inception v3	AUROC: 0.934
Couture et al. [24]	Breast cancer	Molecular subtypes	CBCS3	VGG16	Accuracy: 77%
Sirinukunwattana et al. [25]	Colorectal cancer	Molecular subtypes	FOCUS	Inception v3	AUROC: 0.86~0.92
Hong et al. [26]	Endometrial cancer	Molecular subtypes	TCGA+TCIA	Inception ResNet	AUROC: 0.827~0.934
Sha et al. [27]	Lung cancer	PD-L1 status	Own cohort	ResNet-18	AUROC: 0.80
Schmauch et al. [28]	Various cancers	Expression of multiple genes	TCGA	ResNet-50	Correlation coefficient: ~0.47
He et al. [29]	Breast cancer	Expression of multiple genes	Own cohort	DenseNet-121	Correlation coefficient: ~0.52
Bychkov et al. [30]	Colorectal cancer	Prognosis	HUCH	VGG-16	AUROC: 0.69
Mobadersany et al. [31]	Glioma	Prognosis	TCGA	VGG-19	Harrell’s c index: 0.741
Skrede et al. [32]	Colorectal cancer	Prognosis	AkUH, AUH, GCCS, VICTOR trial	MobileNet v2	Hazard ratio: 3.84
Saillard et al. [33]	Liver cancer	Prognosis	HMUH	ResNet	c index: 0.78
Johannet et al. [34]	Melanoma	Immune checkpoint inhibitors response	NYU	Inception v3	AUROC: 0.691~0.793

**Table 2:** Characteristics of representative pathology foundation models.

Model	Core Architecture	Pretraining Data	Learning Methods
CTransPath	CNN+ multi-scale Swin Transformer	More than 15 million image patches from public datasets including TCGA and PAIP	Semantically relevant contrastive learning (self-supervised learning)
PLIP	ViT-B/32 + text encoder (CLIP framework)	208,414 pathology images paired with natural language descriptions	Image–text contrastive learning to align visual and textual embeddings
HIPT	Cell → Patch → Region hierarchical ViTs (DINO framework)	10,678 WSIs (408,218 4096 × 4096 images, and 104 million 256 × 256 images)	Hierarchical self-supervised representation learning
Virchow	ViT-H (DINOv2 framework)	1,488,550 WSIs derived from 119,629 patients	Self-supervised DINOv2 pretraining (student–teacher knowledge distillation paradigm)
UNI	ViT-L (DINOv2 framework)	More than 100 million images from over 100,000 diagnostic H&E-stained WSIs	Self-supervised DINOv2 pretraining (student–teacher knowledge distillation paradigm)
CHIEF	CTransPath for extracting tile image representations	60,530 WSIs from multiple public and institutional datasets	Unsupervised pretraining on tile images and weakly supervised pretraining on WSIs
GigaPath	DINOv2 for tile representations, autoencoder for slide representations	1.3 billion pathology image tiles from 171,189 WSIs	Slide-level representation learning by LongNet method

**Table 3:** Performance of pathology foundation models across diverse downstream tasks.

Model	Performance of Model on Various Downstream Tasks
CTransPath	Patch retrieval (accuracy = 0.9261), patch classification (accuracy = 0.9820), WSI classification (AUROC = 0.991), mitosis detection (F1 score = 0.7332), and colorectal adenocarcinoma gland segmentation (Dice score = 0.9156)
PLIP	Zero-shot pathology image classification: DigestPath (benign vs. malignant, F1 = 0.832), linear probing: WSSS4LUAD (F1 = 0.927), text-to-image retrieval: PathPedia (Recall@50 = 0.752), image-to-image retrieval: Kather colon (class retrieval, K = 10, accuracy = 0.998)
HIPT	Renal cell carcinoma subtyping (AUROC = 0.980), data-efficient slide classification (low-data regime): renal cell carcinoma subtyping (AUROC = 0.974), survival prediction: clear cell renal cell carcinoma (c-index = 0.642)
Virchow	Pan-cancer detection (9 common + 7 rare, AUROC = 0.950), tissue-specific cancer detection: macrometastasis detection (breast lymph node, AUROC = 0.999), biomarker prediction: breast *CDH1* mutation (AUROC = 0.986)
UNI	43-class OncoTree cancer type classification (AUROC = 0.976), breast cancer metastasis detection (CAMELYON16, AUROC = 0.976), 32-class pan-cancer tissue classification (TCGA, AUROC = 0.975), cell-type segmentation (SegPath): epithelial cells (Dice score = 0.827)
CHIEF	Pan-cancer cancer detection (WSI-level 15 independent datasets/11 cancer types, macro-average AUROC 0.9397), tumour origin identification (AUROC = 0.95-0.99), *IDH* mutation status prediction (glioma, AUROC = 0.9098), MSI status prediction (colorectal cancer, AUROC = 0.875), survival prediction (c-index = 0.74)
GigaPath	Ovarian cancer subtyping (AUROC = 0.98), *EGFR*, *FAT1*, *KRAS*, *TP53* and *LRP1B* mutation prediction in lung cancer (macro-AUROC = 0.626), pan-cancer TMB (AUROC = 0.708), zero-shot cancer subtyping (non-small cell lung cancer, balanced accuracy = 0.64)

**Table 4:** Characteristics of representative multimodal foundation models.

Model	Core Architecture	Pretraining Data	Learning Methods
MedCLIP	Swin Transformer for image encoder + BioClinicalBERT for text encoder	CheXpert (224,316 chest radiographs) and MIMIC-CXR (377,110 chest radiographs)	Image–text alignment with loss based on semantic similarity
BioViL	Hybrid CNN and transformer for image encoder + CXR-BERT for text encoder	MIMIC-CXR v2 chest X-ray with free-text radiology reports dataset (377,110 images)	Image–text contrastive learning to align visual and textual embeddings
PMC-CLIP	Image encoder + text encoder (CLIP style)	1.6 million image-caption pairs collected from PubMed	Image–text contrastive learning + masked language modeling
LLaVA-Rad	BiomedCLIP-CXR as image encoder and Vicuna 7B-v1.5 as text model	697,000 curated radiology image–text pairs	LLaVA style (Instruction-tuned image–text alignment)
RadFM	Image encoder: unified ViT backbone for 2D and 3D images, text encoder: MedLLaMA-13B	15.5 million 2D images and 500,000 3D scans with corresponding captions or diagnosis labels	Instruction-tuned image–text alignment
LLaVA-Med	Image encoder of CLIP + text decoder Vicuna	Large-scale, broad-coverage biomedical figure-caption dataset extracted from PubMed (15 million biomedical image–text pairs)	LLaVA style (Instruction-tuned image–text alignment)
Med-Flamingo	OpenFlamingo-9B	4721 medical textbooks and 1.6 million image-caption pairs collected from PubMed	Cross-attention image–text alignment
PathChat	Vision encoder (ViT-L) + multimodal projector module + LLM (Meta Llama 2)	456,916 instructions with 999,202 questions and answers on pathology	LLaVA style (Instruction-tuned image–text alignment
PRISM2	Image encoder + text encoder → Phi-3 Mini language backbone	2,350,518 WSIs with a corresponding clinical report	Contrastive and autoregressive learning objectives
TITAN	Image encoder + text encoder + multimodal text decoder	335,645 WSIs and 182,862 clinical reports	CoCa (contrastive captioner) method for vision–language alignment
BiomedGPT	Unified encoder–decoder Transformer (BERT-style encoder + GPT-style decoder)	592,567 images, 183 million text sentences, 46,408 object-label pairs and 271,804 image–text pairs	Unified sequence-to-sequence learning with instruction-based multi-task pretraining

**Table 5:** Performance of foundation models across diverse downstream tasks.

Model	Performance of Model on Various Downstream Tasks
MedCLIP	Zero-shot image classification: COVID-19 detection (accuracy = 0.8472), image-to-text retrieval (precision@10 = 0.50)
BioViL	Radiology report generation (CHEXBERT = 31.7 ± 1.0), temporal image classification: pleural effusion (improving/stable/worsening 3-class macro-accuracy = 67.0 ± 0.8%), zero-shot & few-shot pneumonia classification: zero-shot (accuracy = 0.805)/few-shot (accuracy = 0.814)
PMC-CLIP	Zero-shot retrieval: image → text (recall@10 = 71.88%)/text → image (recall@10 = 69.69%), image classification (MedMNIST): PneumoniaMNIST (AUROC = 0.9902), VQA-RAD (accuracy = 77.60%)
LLaVA-Rad	Radiology report generation (factual correctness metric F1-CheXbert-14 score = 57–58), cross-modal retrieval: image → text (recall@10 = 66.53%)/text → image (recall@10 = 67.21%)
RadFM	MRI 2D diagnosis BTM-17 (AUROC = 0.9447), MRI 3D diagnosis BraTS2019 (AUROC = 0.9061), CT 2D diagnosis COVID-CT (AUROC = 0.8137), medical VQA: VQA-RAD (BLEU score = 73.44), report generation (BLEU-3 score = 17.72)
LLaVA-Med	Biomedical visual chat (about 50% compared to GPT-4), medical VQA: SLAKE (accuracy = 87.11%)/VQA-RAD (accuracy = 84.19%)/PathVQA (accuracy = 91.65%)
Med-Flamingo	Medical VQA: VQA-RAD (clinical evaluation score = 5.61)/PathVQA (clinical evaluation score = 2.16)/Visual USMLE (clinical evaluation score = 4.33)
PathChat	Multiple-choice diagnostic question answering: PathQABench-Public (accuracy = 90.5%), open-ended pathology question answering: PathQABench-Public (accuracy = 78.7%), open-ended diagnosis questions (accuracy = 78.5%)
PRISM2	Pan-cancer slide-level detection: 16 tissue origins (AUROC = 0.965)/rare cancers (AUROC = 0.953), breast cancer subtyping (AUROC = 0.979), zero-shot pan-cancer rare cancer detection (balanced accuracy = 0.847)
TITAN	Morphologic cancer subtyping (balanced accuracy = 0.781), molecular classification (balanced accuracy = 0.781), disease-specific survival (c-index = 0.716)
BiomedGPT	Medical VQA: SLAKE (accuracy = 86.1%), pathology VQA: PathVQA (closed-ended accuracy = 88.0%), radiology diagnosis: MC-CXR (accuracy = 89.7%), mammography classification (F1 score ≈ 90%), radiology report summarization (critical error rate: 8.3%)

## Data Availability

No new data were created or analyzed in this study. Data sharing is not applicable to this article.

## Abbreviations

The following abbreviations are used in this manuscript:

AI	Artificial Intelligence
CNN	Convolutional Neural Network
DL	Deep Learning
EBV	Epstein–Barr Virus
H&E	Hematoxylin and Eosin
LLM	large language model
MIL	Multiple-Instance Learning
MSI	Microsatellite Instability
ViT	Vision Transformer
VQA	Visual Question Answering
WSI	Whole-Slide Image
